# Development of an LC–DAD–MS-Based Method for the Analysis of Hydroxyanthracene Derivatives in Food Supplements and Plant Materials

**DOI:** 10.3390/molecules27061932

**Published:** 2022-03-16

**Authors:** Francesca Loschi, Marta Faggian, Stefania Sut, Irene Ferrarese, Erica Maccari, Gregorio Peron, Stefano Dall’Acqua

**Affiliations:** 1 Department of Pharmaceutical and Pharmacological Sciences, University of Padova, Via Marzolo 5, 35131 Padova, Italy; francesca.loschi@studenti.unipd.it (F.L.); stefania.sut@unipd.it (S.S.); irene.ferrarese@unipd.it (I.F.); erica.maccari@unipd.it (E.M.); gregorio.peron@unibs.it (G.P.); 2 Unired srl, Via Niccolo’ Tommaseo 69, 35131 Padova, Italy; nutraceutica@unired.it; 3 Department of Molecular and Translational Medicine, University of Brescia, Viale Europa 11, 25123 Brescia, Italy

**Keywords:** hydroxyanthracene derivatives, LC–DAD–MS, food supplements, botanicals

## Abstract

Products based on plants containing hydroxyanthracene derivatives (HADs)—such as *Rheum*, *Cassia*, and *Aloe* species—are widely used in food supplements or nutraceuticals due to their laxative effects. A more restricted control of HAD contents in food supplements has been implemented by EU Regulation 2021/468, in order to increase the safety of these preparations. Due to their toxicity, aloin A, aloin B, aloe emodin, emodin, and the synthetic derivative danthron have been listed as prohibited substances in food supplements, being tolerated in amounts < 1 mg kg^−1^ in marketed products. In this work, we report the development of a sensitive and fast LC–DAD–MS-based procedure for the determination of these five compounds in food supplements and plant materials or extracts. The entire procedure includes a simple sample preparation step, where target analytes are concentrated by means of solvent extraction and evaporative concentration (solid samples), or by lyophilisation (liquid samples). The average LOQ of 0.10 mg/L, LOD of 0.03 mg/L, accuracy, and precision with CVs below 12.72 were obtained for the studied analytes. This method is suitable for assessing the compliance of commercial products and raw materials with EU Regulation 2021/468. Furthermore, the proposed method can represent a starting point for the development of a unique and standardised analytical approach for the determination of other HADs under the attention of EU authorities.

## 1. Introduction

Hydroxyanthracene derivatives (HADs) are aromatic compounds characterised by a 9,10-dioxoanthracene core, being secondary metabolites widely diffused in the plant kingdom. More than 700 different natural HADs have been reported; 200 are present in flowering plants, and the remainder in lichens and fungi [1]. Plants containing HADs are numerous and belong to different botanical families and genera [2]. HADs are distributed in roots, rhizomes, fruits, flowers, and leaves, where they can be found in free form or conjugated with sugar moieties, with glycosylation being a strategy used by plants to favour their accumulation and storage [3]. Plants containing HADs and isolated HADs are used for their therapeutic properties in medicinal preparations and drugs. Extracts or pulverised plant material from *Rheum* roots, *Senna* leaves, *Polygonum multiflorum*, *Cascara*, *Buckhorn*, and *Aloe* are also used as active ingredients in herbal medicines, food supplements, or “nutraceutical” preparations presenting laxative properties [4,5,6].

Bioactive HADs with different chemical structures and substituents are present at different concentrations in various botanical species. *Rheum* spp. roots and underground parts contain emodin, palmidin C, rhein, sennoside A, and sennoside B, and the concentration of anthraquinones in general ranges from 2.2% to 6.0%, expressed as the % (*w*/*w*) of rhein in the dried plant material. *Cassia* spp. leaves and fruits contain chrysophanol, physcion, and rhein. The content of HAD glycosides in the most used *Cassia senna* L. is in general over 2.5%, expressed as sennoside B [2]. *Cassia senna* L. seeds contain aloe emodin, emodin, emodin anthrone, and physcion, and the amount of HAD glycosides is at least 3.4%. In *Aloe* spp., aloe emodin, aloenin, aloin A, and aloin B are present, and HADs reach a minimum concentration of 18% in the dried drug, expressed as barbaloin [2]. In *Rhamnus frangula* Mil., the minimum content of glucofrangulis in bark is 7.0%, expressed as frangulin A, and in the bark of *Rhamnus purshiana* DC the minimum content of HAD glycosides is 8.0%, expressed as cascaroside A [2].

The European Food Safety Authority (EFSA) noted the effects of a daily dose of 10 mg of HADs from food supplements on the short-term alleviation of occasional constipation [7]. Anticancer, anti-inflammatory, antimicrobial, diuretic, and vasorelaxant properties have also been attributed to HADs [8]; hence, they can find potential applications in the management of several diseases [4,8]. However, these compounds are not devoid of toxicity [9]. Data from a recent work focusing on the hepatotoxicity of emodin, rhein, and aloe emodin suggest that the daily intake levels (231–429 mg day^−1^) and plasma concentrations (7–67 μmol L^−1^) of rhein are associated with toxic effects on HepG2/C3A and HuH-7 cellular models [10]. Another study focusing on the cytotoxicity and genotoxicity of *Aloe vera* whole extract and decolourised extract showed that, after a 24 h treatment, both extracts exhibited concentration-dependent cytotoxicity and mutagenicity in murine lymphoma cells, causing chromosomal mutations and inducing intracellular reactive oxygen species (ROS) levels [11]. Recently, the EFSA provided a scientific opinion on the safety of HADs [2], due to concerns about the possible harmful effects associated with long-term consumption of HAD-containing preparations (i.e., food supplements used as laxatives). In particular, the EFSA [2] reviewed some epidemiological studies that showed an increased risk of colorectal cancer [12,13,14]. In its document, the panel of experts concluded that HADs should be considered to be genotoxic and carcinogenic unless there are specific data demonstrating the contrary [2]. Thus, the EFSA’s opinion could not provide safety advice on the daily intake of products containing hydroxyanthracenes [2]. Therefore, Annex III of Regulation (EC) No. 1925/2006 on the addition of vitamins and minerals and of certain other substances to foods was amended with Commission Regulation (EU) 2021/468, introducing “aloe-emodin, emodin, danthron and all preparations in which these substances are present and preparations from the leaf of *Aloe* species containing hydroxyanthracene derivatives” to Part A (prohibited substances) of the same annex [15]. Consequently, among all HADs, aloe emodin, emodin, and other aloe-related anthraquinones—such as the glycosides aloin A and aloin B—are particularly involved in this recent normative update [16].

According to current EU rules, the contents of aloin A, aloin B, aloe emodin, emodin, and danthron in food supplements cannot exceed 1 mg kg^−1^, i.e., the limit of quantification identified in the summary report of the Standing Committee on “General Food Law” [17]. The inclusion of danthron in this list is unusual. Danthron is a synthetic HAD with a higher genotoxicity than emodin [14], and which could be added to herbal products to enhance their laxative effects [18,19]; furthermore, it can ameliorate dyslipidaemia in the skeletal muscle of obese rats, and attenuate lipid accumulation in HepG2 cells [20]. All of these HADs are used for their laxative effects [20,21], but several studies have also considered some of these compounds for other bioactivities, such as antioxidant, anti-inflammatory, antiproliferative, antidiabetic, and antimicrobial effects [22,23,24,25,26].

Several approaches for the extraction of HADs from natural sources and their chemical characterisation have been presented [24]. The most widely used analytical techniques are liquid chromatography (LC) coupled with UV–Vis detection (comprising diode arrays; DAD), yet useful information and improved selectivity can be attained with mass spectrometry (MS). However, a unique, standardised, and validated method comprising extraction and analysis, suitable for a broad spectrum of food supplements and technical forms (e.g., liquids, juice, tablets, powders), has not yet been defined by regulatory organs. Hence, in this work, we aimed at developing a rapid method for the determination of aloin A, aloin B, aloe emodin, emodin, and danthron in both raw solid and liquid matrices (e.g., vegetal extracts and juices), as well as in commercial products such as food supplements, in order to assess their compliance with EU Regulation 2021/468. To render the method affordable and suitable for a routine use, we developed a simple sample preparation protocol for the extraction and preconcentration of HADs. The method was validated according to the protocol published by the Food and Drug Administration (FDA) for the industry [25].

## 2. Materials and Methods

### 2.1. Solvents and Materials

Aloin A, aloin B, aloe emodin, emodin, and danthron were purchased from Sigma-Aldrich (Milan, Italy). LC-grade acetonitrile, formic acid, and methanol were obtained from Merck KGaA (Darmstadt, Germany). Ultrapure water (18.2 MΩ·cm) was produced using a Milli-Q water purification system from Millipore (Milford, MA, USA). Dry *Cassia senna* L. leaf and fruit extracts (dry extracts A–C), tablets containing extracts of *C. senna* L. and *Aloe*, and liquid products (*Aloe* juices A–C and hydroalcoholic solutions in the form of oral spray and single-dose bottles) with *Aloe* and *C. senna* L. were obtained from external companies. The latter were used to assess the robustness (i.e., the capacity to remain unaffected by small, deliberate variations in method parameters) and sensitivity of the analytical method.

### 2.2. Preparation of Standard Solutions and Samples

Standard stock solutions of reference HADs (aloin A, aloin B, aloe emodin, emodin, and danthron) were prepared by dissolving 1 mg of each compound in 10 mL of methanol. Working standard solutions were prepared by diluting aliquots of each stock solution to obtain eight calibration mixtures in the range of 0.1–20 mg L^−1^ of each analyte.

The procedure for sample preparation from solid matrices was as follows: 1.00 g of dry extract and 250 mg of tablets were ground to obtain a homogeneous powder, which was then extracted with 25 mL of methanol. Extraction was performed in an ultrasound bath for 15 min. The liquid extract was filtered through filter paper and dried under vacuum at 50 °C. The residue was then dissolved in 1.5 mL of methanol.

For liquid samples, 20 g of product was lyophilised, and the freeze-dried material was dissolved in 3 mL of methanol.

### 2.3. Chromatographic Conditions

The system used was an Agilent 1260 binary pump (Agilent Technologies, Santa Clara, CA, USA) equipped with an Eclipse XDB C18 column (4.6 × 250 mm, 5 μm particle size) as the stationary phase, which was maintained at 30 °C. After the column, a passive “T” junction was used to split the flow to the DAD (1260 series) and to a Varian MS-500 Ion Trap Mass Spectrometer (Varian, Santa Clara, CA, USA) equipped with an electrospray ionisation (ESI) source, in order to obtain superimposable chromatograms. A gradient of acetonitrile (A) and Milli-Q water containing 0.1% formic acid (B) was used as the mobile phase. Gradient conditions were optimised in order to perform the analysis in 19 min, and to reach the best separation of aloin A, aloin B, aloe emodin, emodin, and danthron. The gradient was as follows: 0 min, 15% A; 5 min, 35% A; 12 min, 100% A; 18 min, 100% A; 19 min, 15% A. DAD was operated at λ = 350 nm and 425 nm, in order to optimise the detection of aloin A and aloin B, and aloe emodin, emodin, and danthron, respectively.

**Figure 1:**
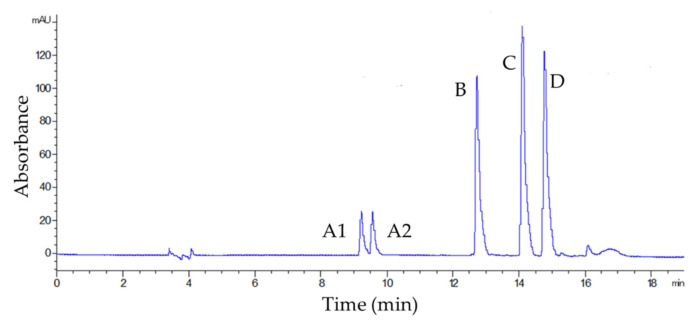
Chromatogram at 254 nm related to analysis of the mix of 10 mg L^−1^ HAD standards aloin B (**A1**), aloin A (**A2**), aloe emodin (**B**), emodin (**C**), and danthron (**D**).

Under the proposed conditions, aloin B was eluted at 9.09 min, aloin A at 9.43 min, aloe emodin at 12.60 min, emodin at 13.95 min, and danthron at 14.62 min (Figure 1).

### 2.4. MS Parameters

**Figure 2:**
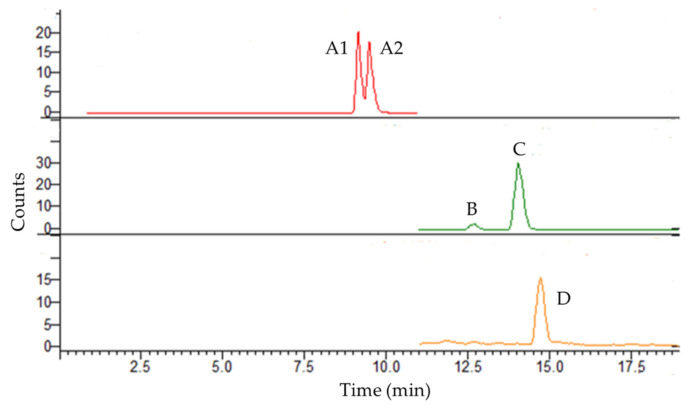
Chromatograms obtained from the LC–MS analysis of aloin B (**A1**) and aloin A (**A2**) (*m*/*z* 417.1), aloe emodin (**B**) and emodin (**C**) (*m*/*z* 269.1), and danthron (**D**) (*m*/*z* 241.1).

MS spectra were acquired using an ESI source. HAD detection was performed in scan mode, selecting the [M − H]^−^ ions at *m*/*z* 417.1 and *m*/*z* 269.2 for aloin A and B, and for aloe emodin and emodin, respectively. Danthron was detected in positive ion mode by selecting the [M + H]^+^ ion with *m*/*z* 241.1. The spectrometer operated in two segments of time using a tandem MS/MS scan in enhanced mode, monitoring the ions corresponding to MS1 and displaying the subsequent fragmentation that was generated. The parameters for the Ion Trap were as follows: needle voltage +5100/−5100; spray shield voltage +600/−600; nebuliser gas pressure 40.0 psi; drying gas pressure 20.0 psi; drying gas temperature 305 °C. For each *m*/*z,* capillary voltage was 90.0 V, and RF loading was 95%. Under the proposed conditions, aloin B was eluted at 9.13 min, aloin A at 9.51 min, aloe emodin at 12.68 min, emodin at 13.07 min, and danthron at 14.76 min (Figure 2).

### 2.5. Method Validation

The method was validated according to the guidelines defined by the FDA for bioanalytical method validation [25]. Briefly, analytical curves were prepared in solvent (methanol) at concentration levels of each analyte ranging from 0.1 to 20 mg L^−1^ (see Section 2.2, plotting peak AUCs vs. concentrations on an XY graph. Recovery of HADs was estimated from solid and liquid matrices. Due to the non-detectable content of HADs, a vegetable matrix composed of *Echinacea purpurea* root was used as dried vegetal material for the recovery test from powder samples. For the recovery test from liquid samples, simple syrup (66.5% sucrose in water) was prepared to simulate the viscosity of *Aloe vera* juices. In both cases, matrices were spiked at three concentration levels (5.00–0.50 mg L^−1^), and HADs were extracted as already described in Section 2.2. Intraday and interday accuracy and precisions were evaluated by analysing spiked matrices at three concentration levels (5.00–0.50 mg L^−1^) three times within the same day, and on two consecutive days, respectively. The precision was represented by the relative standard deviation (RSD %), while the accuracy was represented by the coefficient of variation (CV). The LOD and LOQ were estimated using the analytical curve obtained from a standard solution in a concentration ratio close to the detection limit. The LOD was estimated by using the equation LOD = 3.3 σ/S, and LOQ = 10 σ/S, where σ is the estimate of the SD of the response and S is the slope of the analytical curve.

## 3. Results

**Figure 3:**
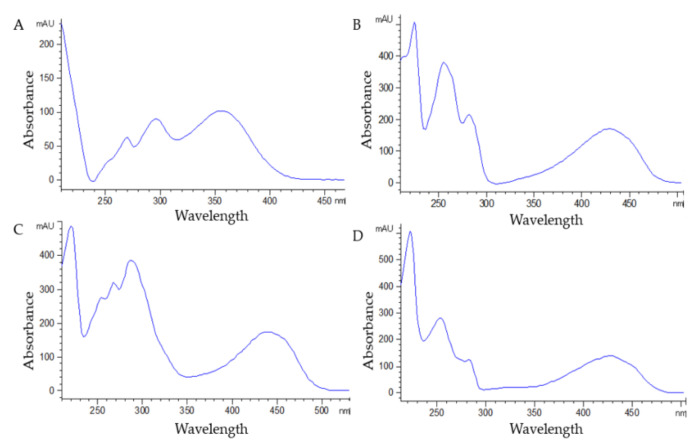
UV–Vis spectra of HAD reference standards: aloin A or aloin B (**A**), aloe emodin (**B**), emodin (**C**), and danthron (**D**).

**Figure 4:**
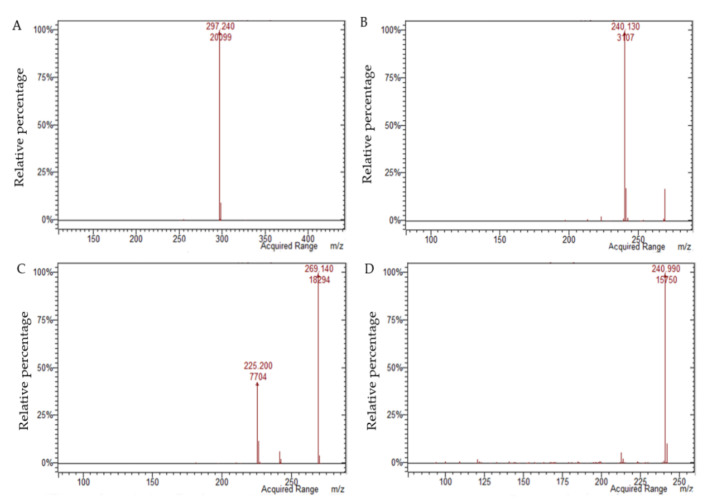
Fragmentation spectra of HAD reference standards obtained from the chromatogram in the MS/MS scan: aloin A or aloin B (**A**), aloe emodin (**B**), emodin (**C**), and danthron (**D**).

The LC–DAD–MS-based method provides information about the presence of HADs (up to mg kg^−1^) in complex matrices and liquid formulations through their characteristic UV–Vis (Figure 3) and ESI mass spectra (Figure 4). The results show also its suitability for the analysis of different types of ingredients for food supplements and nutraceuticals already present on the food market.

The DAD quantification of aloin A and aloin B was performed by monitoring their absorbance at 350 nm, while for aloe emodin, emodin, and danthron, the absorption at 425 nm was considered. These derivatives were detected and quantified in MS according to their main *m*/*z* values and fragments. For the quantification of each analyte in MS, the MS1 signal and those of the most significant fragments in MS2 were considered. For aloin A and B, the latter is the ion at *m*/*z* 297.2, which results from the loss of [C_4_H_8_O_4_] [26]; for aloe emodin it is the ion at *m*/*z* 240.1, generated by the loss of [CHO] [26], along with that at *m*/*z* 241.1, due to the loss of [CO]; for emodin, the ion at *m*/*z* 241.1, due to the loss of [CO], and that at *m*/*z* 225.1, ascribable to the loss of [CHO_2_] [27,28], were considered. For danthron, the ion at *m*/*z* 213.1 generated by the loss of [CO] was considered.

### 3.1. Selectivity, Linearity, LOQ, and LOD

Eight calibration mixtures prepared by mixing different volumes of HAD stock solutions were used to construct analytical curves. The retention time of each HAD reference standard, UV–Vis spectrum, and MS fragmentation spectrum allowed the identification of compounds.

As regards LC–DAD analysis (*n* = 3), all analytical curves showed good linearity in the concentration range of 0.099–21.0 mg L^−1^, with a regression coefficient (r^2^) greater than 0.98. The LOD and LOQ for each HAD reference standard were on average 0.030 and 0.1 µg/mL, respectively. Meanwhile, LC–MS analysis (*n* = 3) showed a polynomial regression equation in the considered calibration range (0.099–10.6 mg L^−1^), with an r^2^ higher than 0.99. The LOD and LOQ for each HAD reference standard were on average 0.030 and 0.1 mg L^−1^, respectively. LC–DAD and LC–MS regression equations are reported in Table 1.

### 3.2. Precision and Accuracy

Analysis of the results in Table 2 reveals that, for the LC–DAD and LC–MS methods, the coefficients of variation varied within the 1.12–12.15 and 0.30–12.72 ranges for intraday and interday precision, respectively. These figures are in compliance with FDA requirements [25].

In Table 3, accuracy in terms of recovery of HAD reference standards in LC–DAD and LC–MS is reported. The amounts of HADs used for spiking ranged from 0.50 to 5.00 μg g^−1^. The mean recovery of HADs in *Echinacea* root and simple syrup obtained via DAD was 92.4% and 97.8%, respectively, while the mean recovery obtained via MS was 100.2% and 95.8%, respectively.

### 3.3. Application

The amounts of HADs detected in dry *C. senna* extracts, as well as in tablets and liquid products containing *C. senna* L. and *Aloe* extracts, are reported in Table 4. These results allow us to assess the effective compliance of the products analysed with Regulation (EU) No. 2021/468 [16], which are potentially available on the market (e.g., aloe juices, hydroalcoholic liquids, and tablets). Regarding dry extracts, the analysis was important in order to determine the amounts of raw materials usable in the finished products.

## 4. Discussion

Plants containing HADs are largely present in herbal medicines and preparations, as well as in food supplements. In 2002, the FDA promulgated a regulation for OTC products containing *A. vera*, and required that all OTC laxative products with *A. vera* should be removed from the US market or reformulated [29].

Since 2021, after the approval of Regulation (EU) No. 2021/468, there has been an increased interest in the development of rapid and sensitive analytical methods for the detection of HADs. There are several published methods for the detection and quantification of HADs, but nobody has been able to quantify the five derivatives present in the European Regulation in a single analysis. The method presented herein was developed to create a unique and standardised approach to comply with the recent Regulation (EU) No. 2021/468, which sets the maximum permissible concentrations for aloin A, aloin B, aloe emodin, emodin, and danthron in food supplements at 1 ppm. For the identification and quantification of HADs, DAD and MS detectors were simultaneously exploited. These compounds have characteristic UV–Vis spectra: aloin A and aloin B have maxima of absorption at 280 nm and 350 nm [30], emodin at 254 nm, 280 nm, and 425 nm, and aloe emodin and danthron at 254 nm and 425 nm [31]. For these reasons, DAD detectors can be used for their quantification. We decided to select the wavelengths at 425 nm for aloe emodin, emodin, and danthron, and 350 nm for aloin A and aloin B, in order to reduce the “noise signal” related to other compounds. The MS analysis was performed in positive ionisation mode for danthron and in negative mode for the other four analytes. The negative mode has already been exploited for the analysis of aloin A [26], aloe emodin [26,32], and emodin [32]. No information could be obtained from the literature for danthron; nevertheless, preliminary results showed that the compound has a better response in the positive ion mode, in comparison to the negative one, and that it presents a worse response in MS compared to the other derivatives.

The LC analysis was conducted in reverse phase, using an Eclipse XDB C18 column that allowed the separation of the five selected compounds with sufficient resolution. The C18 column was selected as the stationary phase, similar to those used for the determination of anthraquinones [33,34]. Recently, a method for the determination of aloin A and B using a Chromolith column was proposed [30], but the present approach has the advantages of involving a more common column, and allowing the separation of the other three involved HADs.

The time required for the chromatographic run is 19 min, and this is a further advantage compared to other methods, which can identify and quantify only two or three anthraquinones in a longer runtime.

For instance, using the methods already present in literature, it is possible to analyse aloe emodin in 10 min [34], aloin A and other few *Aloe* anthrones in 7 min [30], aloe emodin and aloin A in 20 min [33], or aloin and aloe emodin in 42.5 min [33].

Regarding sensitivity, the highest LOQ value for each analyte is 0.09 mg L^−1^, with this concentration being 10 times lower than the highest amount of HADs tolerable in food supplements, as imposed by the EU. Thus, this method allows us to determine the compliance with the European Regulation, with a high grade of certainty. In comparison to the previously published methods, the one proposed herein has the advantage of being able analyse the five HADs at the same time. Some of the previously developed methods are able to quantify HAD concentrations lower (0.05 mg L^−1^) [30] or similar (µg L^−1^) [33] to the present method, but only for a few derivatives at the same time.

The method described herein was evaluated on commercial products and raw materials used as ingredients of nutraceuticals; hence, it can represent a valid aid during the first steps of product development and formulation. In the different samples analysed, danthron was absent, and it was possible to determine the presence of the other HADs. The hydroalcoholic liquid was shown to exceed the aloe emodin and emodin limits, while two of the three juices analysed exceeded the maximum contents of aloin A and B. The tablets showed concentrations of aloin A, aloin B, aloe emodin, and emodin 10–60 times above the limit. Capsules were in line with the EU Regulation, with an amount of each HAD below 1 ppm. The herbal teas A and B had an excess of aloe emodin, with a concentration 10 times higher than the limit. The dry extracts A–D, used as raw materials, were not in line with Regulation (EU) No. 2021/468.

Finally, it must be highlighted that the use of an LC-based method offers an advantage over others based on GC–MS, because it does not require the pre-analytical derivatisation of analytes [35]. To the best of our knowledge, the only GC–MS-based method developed for the screening of four HADs without derivatisation allows their quantification at a lower limit of 3.2 µg mL^−1^, and does not include aloin A and aloin B [36].

## 5. Conclusions

The proposed method is suitable for the determination of aloin B, aloin A, aloe emodin, emodin, and danthron in different kinds of matrices and samples, including solid forms and powders, viscous aqueous juices, and hydroalcoholic liquids; it enables evaluation of the compliance of commercial products with Regulation (EU) No. 2021/468, as well as quantification of the HADs in a short time in solid and liquid matrices, raw materials, and commercial products, with high accuracy and precision. Sample preparation is performed by using commonly diffused laboratory solvents and instrumentation (e.g., Rotavapor and small-scale lyophilizer), and does not require any preparation kit (e.g., SPE cartridges). In addition to being a procedure that can be routinely used in common analytical laboratories, the proposed method can also represent a starting point for the development of a unique and standardised analytical approach for the analysis of other HADs in food matrices, natural extracts, and nutraceuticals.

## Figures and Tables

**Table 1:** Analytical characteristics of the proposed method for HAD standards obtained via LC–DAD and LC–MS (*n* = 3).

Analyte	λ (nm)		Equation	r^2^	Linearity (mg L^−1^)	LOQ (mg L^−1^)	LOD (mg L^−1^)
Aloin B	350		y = 23.133 x − 0.0953	0.9993	0.099–19.8	0.059	0.020
Aloin A	350		y = 16.029 x + 2.0841	0.9866	0.10–20.4	0.060	0.020
Aloe emodin	425		y = 33.222 x + 0.6329	0.9999	0.105–21.0	0.052	0.017
Emodin	425		y = 30.072 x − 1.4086	0.9996	0.105–21.0	0.052	0.017
Danthron	425		y = 29.628 x − 0.4598	0.9989	0.11–21.2	0.082	0.027
**Analyte**	**[M − H]^−^**	**[M + H]^+^**	**Equation**	**r^2^**	**Linearity** **(mg L^−1^)**	**LOQ** **(mg L^−1^)**	**LOD** **(mg L^−1^)**
Aloin B	417.1	na	y = 210.97 x^2^ + 14,693 x	0.9998	0.099–9.9	0.079	0.026
Aloin A	417.1	na	y = 406.57 x^2^ + 13,190 x	0.9993	0.10–10.2	0.080	0.026
Aloe emodin	269.1	na	y = 77.238 x^2^ + 2332.7 x	0.9989	0.105–10.5	0.087	0.029
Emodin	269.1	na	y = −7018.8 x^2^ + 135,531 x	0.9948	0.105–10.5	0.042	0.014
Danthron	na	241.1	y = −555.63 x^2^ + 16,579 x	0.9979	0.11–10.6	0.094	0.031

**Table 2:** Intraday and interday precision and deviation of HADs at different concentrations, measured by LC–DAD and LC–MS.

	Analyte	Nominal Concentration (μg g^−1^)	μg g^−1^ ± SD (CV), LC–DAD	Deviation (%), LC–DAD	μg g^−1^ ± SD (CV), LC–MS	Deviation (%), LC–MS
Intraday precision (*n* = 3)	Aloin B	4.95	4.94 ± 0.08 (1.73)	99.8	5.10 ± 0.09 (1.78)	103.1
		0.99	0.94 ± 0.02 (2.51)	94.9	1.05 ± 0.11 (10.58)	105.7
		0.49	0.43 ± 0.01 (1.9)	87.75	0.49 ± 0.06 (12.15)	98.4
	Aloin A	5.10	4.92 ± 0.11 (1.97)	96.5	4.98 ± 0.07 (1.44)	97.7
		1.02	0.98 ± 0.04 (4.45)	96.1	1.01 ± 0.12 (11.99)	99.4
		0.51	0.48 ± 0.005 (1.12)	94.1	0.48 ± 0.04 (8.98)	93.5
	Aloe emodin	5.25	5.34 ± 0.12 (2.18)	101.7	5.07 ± 0.44 (8.61)	96.6
		1.05	1.01 ± 0.02 (2.43)	96.2	1.05 ± 0.06 (5.58)	100.1
		0.52	0.53 ± 0.01 (1.98)	101.9	0.49 ± 0.05 (9.49)	93.7
	Emodin	5.25	5.26 ± 0.09 (1.83)	100.2	5.22 ± 0.08 (1.57)	99.1
		1.05	1.02 ± 0.02 (1.83)	97.1	1.04 ± 0.09 (8.62)	99.2
		0.52	0.51 ± 0.02 (3.95)	98.1	0.60 ± 0.03 (5.43)	108.2
	Danthron	5.30	5.45 ± 0.16 (2.89)	102.8	5.35 ± 0.33 (6.2)	100.9
		1.06	0.98 ± 0.03 (3.39)	92.4	0.99 ± 0.07 (6.75)	93.1
		0.53	0.52 ± 0.01 (1.55)	98.1	0.49 ± 0.04 (8.44)	92.8
Interday precision (*n* = 3)	Aloin B	4.95	4.96 ± 0.06 (1.29)	99.8	5.08 ± 0.06 (1.21)	102.7
		0.99	0.93 ± 0.05 (5.87)	93.9	0.92 ± 0.07 (8.05)	92.5
		0.49	0.44 ± 0.02 (3.95)	88.9	0.47 ± 0.03 (7.44)	94.9
	Aloin A	5.10	4.92 ± 0.11 (1.97)	96.5	4.95 ± 0.10 (2.06)	97.2
		1.02	0.98 ± 0.04 (4.45)	96.1	1.11 ± 0.02 (1.59)	109.0
		0.51	0.46 ± 0.005 (1.15)	90.2	0.54 ± 0.04 (8.18)	105.6
	Aloe emodin	5.25	5.53 ± 0.34 (5.96)	105.3	5.30 ± 0.10 (1.94)	101.0
		1.05	1.00 ± 0.02 (2.43)	95.2	1.01 ± 0.11 (11.19)	96.4
		0.52	0.52 ± 0.01 (2.08)	99.0	0.51 ± 0.02 (4.62)	96.8
	Emodin	5.25	5.48 ± 0.21 (3.82)	104.2	5.05 ± 0.31 (6.18)	96.0
		1.05	1.06 ± 0.03 (2.82)	100.9	1.04 ± 0.08 (7.78)	98.6
		0.52	0.56 ± 0.01 (1.55)	105.7	0.59 ± 0.06 (9.47)	111.5
	Danthron	5.30	5.49 ± 0.11 (1.96)	103.6	5.52 ± 0.08 (1.5)	104.2
		1.06	0.96 ± 0.06 (5.94)	90.6	0.94 ± 0.003 (0.3)	88.8
		0.53	0.51 ± 0.02 (3.89)	96.2	0.51 ± 0.06 (12.72)	95.8

Concentration values are reported as μg g^−1^ ± standard deviations (SD). CV indicates the coefficient of variation.

**Table 3:** Recovery of HAD reference standards added to *Echinacea* extract and simple syrup in LC–DAD and LC–MS.

Sample	HAD	Spiking Concentration (μg g^−1^)	μg g^−1^ ± SD, LC–DAD	Recovery (%), LC–DAD	μg g^−1^ ± SD, LC–MS	Recovery (%), LC–MS
*Echinacea* root + HADs (*n* = 3)	Aloin B	4.95	4.28 ± 0.10	86.5	5.03 ± 0.11	101.6
	Aloin A	5.10	4.72 ± 0.08	92.5	5.55 ± 0.16	108.8
	Aloe emodin	5.25	5.69 ± 0.12	108.4	5.92 ± 0.23	112.8
	Emodin	5.26	4.66 ± 0.13	88.6	5.09 ± 0.12	96.8
	Danthron	5.30	4.80 ± 0.14	90.6	4.55 ± 0.15	85.8
*Echinacea* root + HADs (*n* = 3)	Aloin B	0.99	0.91 ± 0.04	91.9	1.01 ± 0.08	102.0
	Aloin A	1.02	0.97 ± 0.03	95.1	1.21 ± 0.15	118.6
	Aloe emodin	1.05	1.18 ± 0.06	112.4	1.14 ± 0.12	108.6
	Emodin	1.05	0.90 ± 0.04	85.7	0.86 ± 0.12	81.9
	Danthron	1.06	0.97 ± 0.02	91.5	0.90 ± 0.11	84.9
*Echinacea* root + HADs (*n* = 3)	Aloin B	0.51	0.42 ± 0.01	82.3	0.51 ± 0.05	100.0
	Aloin A	0.49	0.43 ± 0.02	87.7	0.58 ± 0.06	118.4
	Aloe emodin	0.52	0.57 ± 0.03	91.2	0.57 ± 0.07	109.6
	Emodin	0.53	0.44 ± 0.03	83.0	0.45 ± 0.03	84.9
	Danthron	0.53	0.47 ± 0.02	88.7	0.47 ± 0.07	88.7
Simple syrup + HADs (*n* = 3)	Aloin B	4.95	4.47 ± 0.11	90.3	4.76 ± 0.15	96.2
	Aloin A	5.10	4.70 ± 0.12	92.1	5.42 ± 0.12	106.3
	Aloe emodin	5.25	5.69 ± 0.16	108.4	5.83 ± 0.15	111.0
	Emodin	5.26	5.56 ± 0.09	105.7	4.87 ± 0.12	92.6
	Danthron	5.30	5.45 ± 0.13	102.8	5.32± 0.12	100.4
Simple syrup + HADs (*n* = 3)	Aloin B	0.99	0.92 ± 0.07	92.9	0.93 ± 0.11	93.9
	Aloin A	1.02	0.92 ± 0.05	89.3	1.15 ± 0.07	112.7
	Aloe emodin	1.05	1.10 ± 0.04	104.8	0.89 ± 0.09	84.8
	Emodin	1.05	1.14 ± 0.06	108.6	0.97 ± 0.03	92.4
	Danthron	1.06	1.07 ± 0.03	100.9	0.93 ± 0.06	87.7
Simple syrup + HADs (*n* = 3)	Aloin B	0.51	0.44 ± 0.04	86.3	0.45 ± 0.05	88.2
	Aloin A	0.49	0.42 ± 0.02	85.7	0.49 ± 0.04	100.0
	Aloe emodin	0.52	0.44 ± 0.04	84.6	0.46 ± 0.03	88.5
	Emodin	0.53	0.63 ± 0.05	118.9	0.48 ± 0.06	90.6
	Danthron	0.53	0.51 ± 0.01	96.2	0.49 ± 0.06	92.4

**Table 4:** HADs detected in different samples (*n* = 3).

Sample	Aloin B	Aloin A	AloeEmodin	Emodin	Danthron	
Hydroalcoholic liquid single-dose bottles	nd	nd	2.08 ± 0.09	1.04 ± 0.03	nd	
Hydroalcoholic liquidoral spray	nd		nd	10.39 ± 1.66	0.60 ± 0.025	nd
*Aloe vera* juice A	0.26 ± 0.02		0.81 ± 0.10	nd	nd	nd
*Aloe vera* juice B	8.19 ± 2.04		11.21 ± 2.82	nd	nd	nd
*Aloe vera* juice C	0.04 ± 0.01		0.06 ± 0.01	nd	nd	nd
Tablets	27.08 ± 3.23		32.98 ± 2.79	63.96 ± 0.41	9.88 ± 0.01	nd
Capsules	nd	nd	nd	nd	nd	
Herbal tea A	nd	nd	9.78 ± 0.88	0.31 ± 0.04	nd	
Herbal tea B	nd	nd	10.36 ± 0.92	0.13 ± 0.04	nd	
Dry extract A	nd	nd	3.23 ± 0.19	1.24 ± 0.08	nd	
Dry extract B	nd	nd	nd	1.13 ± 0.11	nd	
Dry extract C	nd	nd	1.85 ± 0.17	0.62 ± 0.21	nd	
Dry extract D	nd	nd	65.49 ± 4.26	64.57 ± 7.76	nd	

nd: not detected (<LOD). The amount of each derivative is reported in mg/kg.

## Data Availability

All data generated or analysed during this study are included in this published article.

## Sample Availability

Samples of the compounds are available from the authors.

## References

[1] Gonfa Y.H., Beshah F., Tadesse M.G., Bachheti A., Bachheti R.K. (2021). Phytochemical Investigation and Potential Pharmacologically Active Compounds of Rumex Nepalensis: An Appraisal. J. Basic Appl. Sci..

[2] Younes M., Aggett P., Aguilar F., Crebelli R., Filipič M., Frutos M.J., Galtier P., Gott D., Gundert-Remy U., EFSA Panel on Food Additives and Nutrient Sources added to Food (ANS) (2018). Safety of Hydroxyanthracene Derivatives for Use in Food. EFSA J..

[3] Pandith S., Hussain A., Bhat W., Dhar N., Qazi A., Rana S., Razdan S., Wani T., Shah M., Bedi Y.S. (2014). Evaluation of Anthraquinones from Himalayan Rhubarb (Rheum Emodi Wall. Ex Meissn.) as Antiproliferative Agents. S. Afr. J. Bot..

[4] Malik E.M., Müller C.E. (2016). Anthraquinones As Pharmacological Tools and Drugs. Med. Res. Rev..

[5] Lombardi N., Bettiol A., Crescioli G., Maggini V., Gallo E., Sivelli F., Sofi F., Gensini G.F., Vannacci A., Firenzuoli F. (2020). Association between Anthraquinone Laxatives and Colorectal Cancer: Protocol for a Systematic Review and Meta-Analysis. Syst. Rev..

[6] Mascolo N., Capasso R., Capasso F. (1998). Senna. A Safe and Effective Drug. PTR.

[7] EFSA Panel on Dietetic Products, Nutrition and Allergies (NDA) (2013). Scientific Opinion on the Substantiation of a Health Claim Related to Hydroxyanthracene Derivatives and Improvement of Bowel Function Pursuant to Article 13(5) of Regulation (EC) No 1924/2006. EFSA J..

[8] Chien S.-C., Wu Y.-C., Chen Z.-W., Yang W.-C. (2015). Naturally Occurring Anthraquinones: Chemistry and Therapeutic Potential in Autoimmune Diabetes. Evid. Based Complement. Altern. Med..

[9] Guo X., Mei N. (2016). *Aloe vera*: A Review of Toxicity and Adverse Clinical Effects. J. Environ. Sci. Health C.

[10] Liu Y., Mapa M., Sprando R. (2020). Liver Toxicity of Anthraquinones: A Combined in Vitro Cytotoxicity and in Silico Reverse Dosimetry Evaluation. Food Chem. Toxicol..

[11] Guo X., Zhang S., Dial S.L., Boudreau M.D., Xia Q., Fu P.P., Levy D.D., Moore M.M., Mei N. (2014). In Vitro Investigation of the Mutagenic Potential of *Aloe vera* Extracts. Toxicol. Res..

[12] Siegers C.-P., Siemers J., Baretton G. (1993). Sennosides and Aloin Do Not Promote Dimethylhydrazine-Induced Colorectal Tumors in Mice. PHA.

[13] Nusko G., Schneider B., Müller G., Kusche J., Hahn E.G. (1993). Retrospective Study on Laxative Use and Melanosis Coli as Risk Factors for Colorectal Neoplasma. PHA.

[14] Sonnenberg A., Müller A.D. (1993). Constipation and Cathartics as Risk Factors of Colorectal Cancer: A Meta-Analysis. PHA.

[15] EUR-Lex 32006R1925 EN EUR-Lex Regulation (EC) No 1925/2006 of the European Parliament and of the Council of 20 December 2006. https://eur-lex.europa.eu/eli/reg/2006/1925.

[16] EUR-Lex 32021R0468 EN EUR-Lex Commission Regulation (EU) 2021/468 of 18 March 2021. https://eur-lex.europa.eu/eli/reg/2021/468/oj.

[17] Standing Committee on Plants, Animals, Food and Feed Section General Food Law. 5 October 2020. Exchange of Views and Possible Opinion of the Committee on a Draft Commission Regulation (EU) Amending Annex III to Regulation (EC) No 1925/2006 of the European Parliament and of the Council as Regards Botanical Species Containing Hydroxyanthracene Derivatives.Sante.Ddg2.g.5(2020)7913268. https://www.aade.gr/sites/default/files/2021-06/reg-com_gfl_20201005_sum_0.pdf.

[18] Müller S.O., Eckert I., Lutz W.K., Stopper H. (1996). Genotoxicity of the Laxative Drug Components Emodin, Aloe-Emodin and Danthron in Mammalian Cells: Topoisomerase II Mediated?. Mutat. Res. Genet. Toxicol. Environ. Mutagen..

[19] Metzger W., Reif K. (1996). Determination of 1,8-Dihydroxyanthranoids in Senna. J. Chromatogr. A.

[20] Shi X., Zhang Y., Lin B., Zhou Y., Suo W., Wei J., Zhang L., Lin J., Xiao F., Zhao L. (2021). Danthron Attenuates Experimental Atherosclerosis by Targeting Foam Cell Formation. Exp. Physiol..

[21] Dong X., Fu J., Yin X., Cao S., Li X., Lin L., Ni J., Huyiligeqi (2016). Emodin: A Review of Its Pharmacology, Toxicity and Pharmacokinetics. PTR.

[22] Harlev E., Nevo E., Lansky E.P., Ofir R., Bishayee A. (2012). Anticancer Potential of Aloes: Antioxidant, Antiproliferative, and Immunostimulatory Attributes. Planta Med..

[23] Salehi B., Albayrak S., Antolak H., Kręgiel D., Pawlikowska E., Sharifi-Rad M., Uprety Y., Tsouh Fokou P.V., Yousef Z., Amiruddin Zakaria Z. (2018). *Aloe* Genus Plants: From Farm to Food Applications and Phytopharmacotherapy. Int. J. Mol. Sci..

[24] Duval J., Pecher V., Poujol M., Lesellier E. (2016). Research Advances for the Extraction, Analysis and Uses of Anthraquinones: A Review. Ind. Crops Prod..

[25] Food and Drug Administration, Center for Drug Evaluation and Research (CDER), Center for Veterinary Medicine (CMV) (2018). Bioanalytical Method Validation Guidance for Industry.

[26] Wang P.G., Zhou W., Wamer W.G., Krynitsky A.J., Rader J.I. (2012). Simultaneous Determination of Aloin A and Aloe Emodin in Products Containing *Aloe vera* by Ultra-Performance Liquid Chromatography with Tandem Mass Spectrometry. Anal. Methods.

[27] Koyama J., Takeuchi A., Morita I., Nishino Y., Shimizu M., Inoue M., Kobayashi N. (2009). Characterization of Emodin Metabolites in Raji Cells by LC–APCI-MS/MS. Bioorg. Med. Chem..

[28] Fan J.J., Li C.H., Hu Y.J., Chen H., Yang F.Q. (2018). Comparative Assessment of In Vitro Thrombolytic and Fibrinolysis Activity of Four Aloe Species and Analysis of Their Phenolic Compounds by LC–MS. S. Afr. J. Bot..

[29] Food and Drug Administration (2002). HHS Status of Certain Additional Over-the-Counter Drug Category II and III Active Ingredients. Final Rule. Fed. Regist..

[30] Sibhat G., Kahsay G., Van Schepdael A., Adams E. (2021). Fast and Easily Applicable LC-UV Method for Analysis of Bioactive Anthrones from Aloe Leaf Latex. J. Pharm. Biomed. Anal..

[31] Kline D., Ritruthai V., Babajanian S., Gao Q., Ingle P., Chang P., Swanson G. (2017). Quantitative Analysis of Aloins and Aloin-Emodin in *Aloe vera* Raw Materials and Finished Products Using High-Performance Liquid Chromatography: Single-Laboratory Validation, First Action 2016.09. J. AOAC Int..

[32] Zhang L., Ma W., Li J., He J., Zhang P., Zheng F., Zhang B., Gao X., Chang Y. (2013). Influence of Processing on Pharmacokinetic of Typical Constituents in Radix Polygoni Multiflori after Oral Administration by LC–ESI–MS/MS. J. Ethnopharmacol..

[33] Elsohly M.A., Gul W., Avula B., Khan I.A. (2007). Determination of the Anthraquinones Aloe-Emodin and Aloin-A by Liquid Chromatography with Mass Spectrometric and Diode Array Detection. J. AOAC Int..

[34] Mandrioli R., Mercolini L., Ferranti A., Fanali S., Raggi M. (2011). Determination of Aloe Emodin in *Aloe vera* Extracts and Commercial Formulations by HPLC with Tandem UV Absorption and Fluorescence Detection. Food Chem..

[35] Zhu Z., Li J., Gao X., Amponsem E., Kang L., Hu L., Zhang B., Chang Y. (2012). Simultaneous Determination of Stilbenes, Phenolic Acids, Flavonoids and Anthraquinones in Radix Polygoni Multiflori by LC–MS/MS. J. Pharm. Biomed. Anal..

[36] Dai H., Chen Z., Shang B., Chen Q. (2018). Identification and Quantification of Four Anthraquinones in Rhubarb and Its Preparations by Gas Chromatography-Mass Spectrometry. J. Chromatogr. Sci..

